# Mortality in Community-Acquired Sepsis and Infections in the Faroe Islands—A Prospective Observational Study

**DOI:** 10.3390/idr16030033

**Published:** 2024-05-13

**Authors:** Marija Todorovic Markovic, Mirjana Todorovic Mitic, Aleksandra Ignjatovic, Magnús Gottfredsson, Shahin Gaini

**Affiliations:** 1 Department of Cardiology, Copenhagen University Hospital-Rigshospitalet, 2100 Copenhagen, Denmark; 2 Department of Infectious Diseases, Odense University Hospital, 5000 Odense, Denmark; 3 Department of Medicine, Infectious Diseases Division, National Hospital of the Faroe Islands, JC. Svabosgøta 41-49, 100 Torshavn, Faroe Islands; 4 Clinic of Oncology, Clinical Centre, 18000 Nis, Serbia; 5 Department of Medical Statistics and Informatics, School of Medicine, University of Nis, 18108 Nis, Serbia; 6 Department of Infectious Diseases, Landspitali University Hospital, 105 Reykjavik, Iceland; 7 Faculty of Medicine, School of Health Sciences, University of Iceland, 101 Reykjavik, Iceland; 8 Faculty of Health Sciences, University of the Faroe Islands, 100 Torshavn, Faroe Islands

**Keywords:** community-acquired infection, sepsis, mortality

## Abstract

The aim of this study was to collect data and analyze mortality among patients hospitalized with community-acquired infections in the Faroe Islands. A prospective observational study was conducted in the Medical Department of the National Hospital of the Faroe Islands from October 2013 to April 2015. Cumulative all-cause, in-hospital, short-term, intermediate-term and long-term mortality rates were calculated. Kaplan–Meier survival curves comparing infection-free patients with infected patients of all severities and different age groups are presented. A log-rank test was used to compare groups. Mortality hazard ratios were calculated for subgroups using Cox regression multivariable models. There were 1309 patients without infection and 755 patients with infection. There were 51% female and 49% male patients. Mean age was 62.73 ± 19.71. Cumulative all-cause mortality and in-hospital mortality were highest in more severe forms of infection. This pattern remained the same for short-term mortality in the model adjusted for sex and age, while there were no significant differences among the various infection groups in regard to intermediate- or long-term survival after adjustment. Overall and short-term mortality rates were highest among those with severe manifestations of infection and those with infection compared to infection-free patients.

## 1. Introduction

Sepsis is a common, often deadly and cost-demanding disease worldwide [1]. According to Vincent et al., sepsis mortality rates rank above some of the other leading causes of in-hospital deaths, such as stroke and acute myocardial infarction [2]. The mortality rates of community-acquired sepsis of any severity, severe sepsis and septic shock vary between different studies depending on the origin of the infection, the severity of sepsis and the observation period [3,4,5,6,7,8,9,10,11].

On the local level, epidemiological knowledge regarding infectious diseases, including microbiology, risk populations, severity of disease and other clinical characteristics, is essential to tailoring local guidelines for diagnosing and treating infection. Baseline knowledge about outcomes is needed to monitor changes in outcomes over time, including changes that might be due to changes in population characteristics (examples: age, vaccination policies, use of immunosuppressives, comorbidity in the population, access to health care, general health status of the population and other aspects), changes in treatment, or both. At the moment, there is very scarce knowledge about infectious disease mortality in the Faroe Islands. The aim of this study was to investigate outcomes, measured as the mortality rate, among hospitalized patients with community-acquired infections in the Faroe Islands. 

## 2. Materials and Methods

### 2.1. Study Design and Setting

This research study is based on data from an epidemiological sepsis study conducted in the Faroe Islands from 2013 to 2015. All medical adult patients were included in a prospective manner when they were admitted to the largest hospital in the Faroe Islands. Two previous publications have described in detail the study design, inclusion criteria, exclusion criteria and study definitions regarding the patients included in the analyses of this paper [12,13]. In short, all newly admitted medical adult patients in the 18-month study period from 2013 to 2015 were included in this prospective observational study. The National Hospital of the Faroe Islands has a catchment area of approximately 78% of the whole Faroese population [14]. This makes the study a nearly nation-wide study. Rigorous definitions were used to classify patients as having infection, sepsis, severity of sepsis, or not having infection as their cause of admission [12,13]. For detailed aspects of the methodology of the prospective observational study on sepsis epidemiology, focus of infection and etiology of infection, we refer to the previously published papers [12,13]. The current study presented in this paper is focused on mortality aspects, while the two previously published papers focused on epidemiology, cause of infection and focus of infection [12,13].

This study was planned in 2012 and started in 2013. At that time, the SIRS (Systemic Inflammatory Response Syndrome) criteria were the official criteria used in sepsis studies [12,15]. In 2016, the Third International Consensus Definitions for Sepsis and Septic Shock (Sepsis-3) [15] were published. We decided to keep our sepsis criteria as originally defined in the protocol.

### 2.2. Definition of the Infection-Free Cohort and the Infection Cohort

For patients admitted to hospital more than once during the study period, only one episode was used for inclusion in one of the cohorts.

The infection-free cohort included patients having at least one admission without infection and no admissions with infection during the study period. For patients with more than one admission without infection, the last episode was included.

The infection cohort included patients having at least one admission with infection during the study period. For patients with more than one admission with infection, the episode with the most severe stage according to the SIRS and severity criteria was included (infection without sepsis < sepsis without severe sepsis or septic shock < severe sepsis < septic shock). If a patient was admitted more than once with an infection with the same severity, the last episode was included. 

### 2.3. Data Analyses

Data were presented as means and standard deviation (absolute and relative values). We divided follow-up into short term (0–28 days), intermediate term (29–180 days) and long term (181+ days). Follow-up stopped at the 5th of October 2016. Cumulative all-cause mortality proportions were calculated for infection-free patients, patients with infection without sepsis, patients with sepsis without severe sepsis or septic shock, patients with severe sepsis, patients with septic shock and patients with sepsis of any severity. Ninety-five percent CIs were calculated under the assumption of a Poisson distribution. The mortality data among patients hospitalized with infection without sepsis, patients with sepsis without severe sepsis or septic shock, patients with severe sepsis and patients with septic shock were presented in Kaplan–Meier curves. A log-rank test was used to compare groups. Two Cox proportional hazard regression models were used in relation to different groups of infection and sepsis: (a) an unadjusted analysis and (b) a multivariable analysis adjusted for age and gender. Because of missing values in the infection-free group, regarding the Charlson comorbidity index [16], we could not present a model adjusted for comorbidities. The group of patients without infection was used as a reference group. The multivariable Cox regression analysis was not carried out in the septic shock category for intermediate-term mortality because of the small number of patients. We calculated hazard ratios (HRs) for every follow-up group. All statistical analyses were performed using R software packages version 3.1.2 [17].

## 3. Results

### 3.1. Patient Characteristics

There were 3615 admissions in the study period. After exclusion of admissions with hospital-acquired infection and transfers from other hospitals, our study group counted 1054 admissions with a community-acquired infection of any severity and 2302 admissions without infection. There were 1309 patients in the infection-free group and 755 patients in the group with infection, including 298 patients (39% of all patients with infection) in the group with infection without sepsis, 214 patients (28% of all patients with infection) in the group with sepsis without severe sepsis or septic shock, 223 patients (30% of all patients with infection) in the group with severe sepsis and 20 patients (3% of all patients with infection) in the septic shock group. There was a slight difference in the number of male and female patients, with 51% female and 49% male patients. Mean age was 62.73 ± 19.71 (range: 16–102 years). Detailed demographic and clinical characteristics are presented in Table 1.

### 3.2. All-Cause and in-Hospital Mortality

Overall cumulative all-cause mortality was highest in patients with septic shock, followed by patients with severe sepsis. There were significant differences between groups (*p* < 0.001) (Table 2 and Table 3).

In an unadjusted model, patients with septic shock had the highest HR for all-cause mortality (HR 27.521, 95%CI 16.136–46.023), followed by patients with severe sepsis (HR 6.438, 95%CI 4.949–8.375), patients with infection without sepsis (HR 4.452, 95%CI 3.431–5.778) and patients with sepsis without severe sepsis or septic shock (HR 3.807, 95%CI 2.827–5.128). In the adjusted model, the pattern remained the same, with the exception that patients with sepsis without severe sepsis or septic shock had a slightly higher HR than patients with infection without sepsis (Table 4).

In-hospital mortality was highest in patients with septic shock and lowest in patients without infection. There were significant differences between the groups (*p* < 0.001) (Table 2).

**Figure 1:**
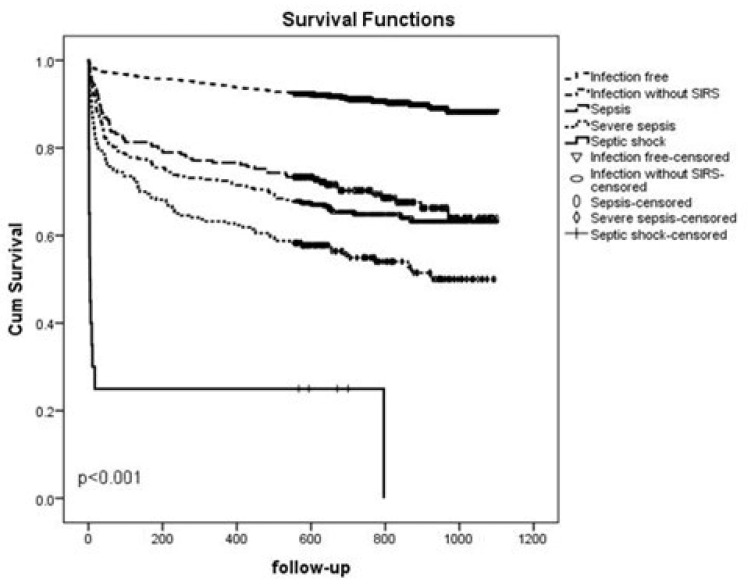
Kaplan–Meier survival curve for patients hospitalized over an 18-month period in the Medical Department without infection, with infection without SIRS, with sepsis, severe sepsis and septic shock.

Kaplan–Meier curves showed that patients with septic shock had the shortest survival. Our results showed significant differences between infection in groups with different severities (*p* < 0.001) (Figure 1).

**Figure 2:**
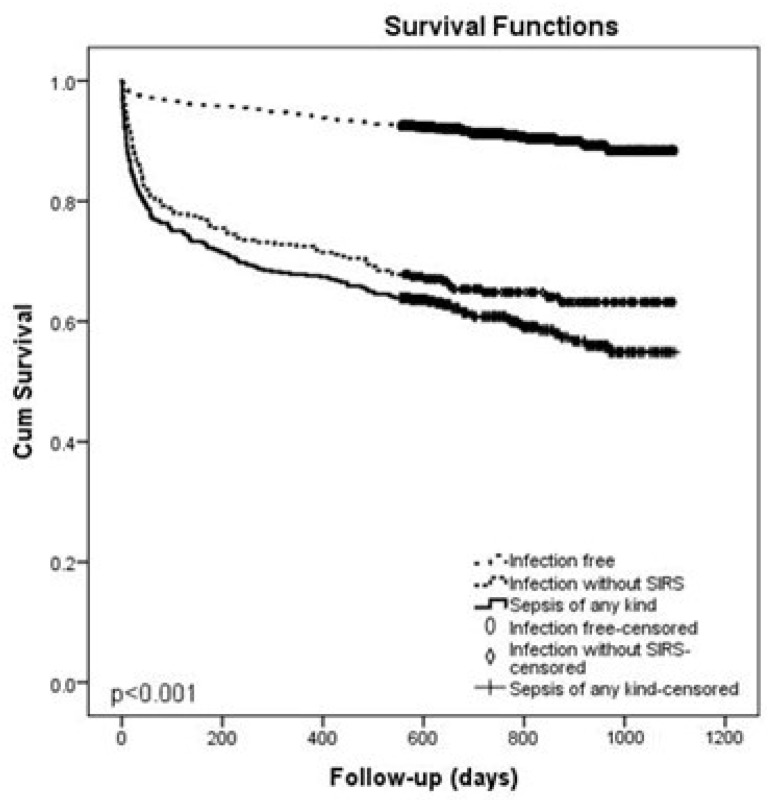
Kaplan–Meier survival curves comparing patients without infection, infection without SIRS and sepsis of any severity.

Patients with sepsis had significantly shorter survival than patients without infection (*p* < 0.001) (Figure 2).

**Figure 3:**
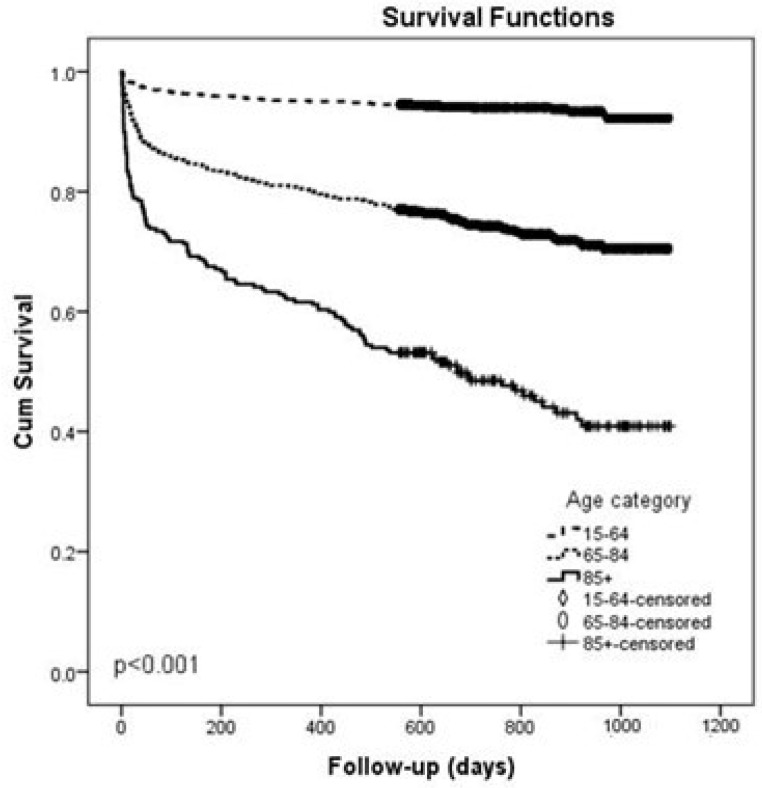
Kaplan–Meier curves of one-year survival for patients with sepsis of any severity, comparing age categories.

The oldest patients with sepsis of any severity had the shortest long-term survival. Our results showed significant differences between age categories in survival after sepsis of any severity (*p* < 0.001) (Figure 3).

### 3.3. Short-Term Mortality

Our results showed that the 28-day mortality was highest among patients with severe sepsis and septic shock (*p* < 0.001). All groups with infection had significantly higher 28-day mortality rates compared to infection-free patients (*p* < 0.001 for all groups). The 28-day mortality was highest in patients with septic shock (75.0%, 95%CI 50.1–99.9) and in patients with severe sepsis (20.2%, 95%CI 14.7–27.0). The 28-day mortality for patients with sepsis without severe sepsis or septic shock was 9.3% (95%CI 5.7–14.4).

Patients with septic shock had the highest HR for short-term mortality (HR 71.724, 95%CI 38.110–134.98), followed by patients with severe sepsis (HR 10.366, 95% CI 6.466–16.616), patients with infection without sepsis (HR 6.052, 95% CI 3.704–9.889) and patients with sepsis without severe sepsis or septic shock (HR 4.486, 95% CI 2.527–7.962). In the model adjusted for age and gender, the differences between infection without sepsis and sepsis without severe sepsis or septic shock evened out (Table 4).

### 3.4. Intermediate-Term Mortality

The HR for the time period of 31–180 days was significantly higher in patients with infection than in infection-free patients in both unadjusted and adjusted models. The HRs for patients with infection without sepsis and patients with severe sepsis were almost equal in the unadjusted model. The HR for septic shock was not calculated due to the small number of patients. In the adjusted model, differences between all three infection groups evened out, remaining over 5 (Table 4).

### 3.5. Long-Term Mortality

In an unadjusted model and model adjusted for gender and age, all infection severities except septic shock were predictors for long-term mortality. In an unadjusted model, the HR for long-term mortality was highest in patients with severe sepsis (Table 4).

## 4. Discussion

### 4.1. Principal Findings

Our results showed that the existence of infection, and most specifically more severe forms of infection, significantly influenced the short-term outcome after a sepsis episode. As expected, survival after such an episode was shorter in the older population.

### 4.2. Strengths

The major strengths of this study are the prospective design, inclusion throughout 1.5 years and manual screening of every admitted patient in the study period. This type of inclusion allowed us to find all patients with infection and to exclude all values that could be influenced by other comorbidities and other acute conditions. Furthermore, we included patients with an infection of any severity, not only patients with sepsis.

### 4.3. Limitations

A limitation is that we limited our study population to the patients admitted to the Medical Department and to medical patients from the ICU. We did not include patients from the other two hospitals in the Faroe Islands, nor patients admitted to the Surgical Department at the National Hospital of the Faroe Islands. Some of the patients were admitted multiple times. As mortality analyses required the number of patients and not the number of admissions, we chose to use a single admission with the most severe infection when patients had been admitted several times within the study period with infections of varying degrees of severity. This selection bias is a second limitation. In this way, we tried to estimate the “true” survival rate of severe sepsis but might have underestimated the mortality in other groups.

Another limitation in our study is that patients were included in the period 2013 to 2015, almost 10 years ago. This study, as an observational prospective study, was very time-consuming in data retrieval. Data analyses were finished in 2019, and a PhD study based on the study finished in 2019. As infectious disease doctors, and because of the COVID pandemic in 2020–2022, the present study was not prioritized until 2023 because of demanding clinical duties in handling patients with COVID in the Faroe Islands and the focus of our group on COVID research in the period 2020–2023. The data must therefore be interpretated in the context of this time delay.

### 4.4. Comparison to Other Studies

We found that the severity of infection was a strong independent predictor of all-cause, short-term and long-term mortality and that patients with septic shock had the shortest survival. Our results are in line with some studies but differ from others. The 28-day mortality in our study, in all infection severities, was higher than that in the study by Davis et al. [5]. When compared to the study by Rodríguez et al., our 28-day mortality of sepsis of any severity was similar to their study and our mortality of septic shock was higher, but the mortalities of sepsis without severe sepsis or septic shock and severe sepsis were lower in our study [18]. However, our cumulative 28-day mortality was higher than that reported in the article from Henriksen et al. [11], but the differences between the groups were in line with their results.

We found that in-hospital and short-term mortality were significantly higher in patients with infection in comparison to infection-free patients admitted to the Medical Department. Mortalities were highest in patients with septic shock and severe sepsis. In-hospital mortality of severe sepsis was, in our study, higher compared to mortality in medical patients with community-acquired severe sepsis in the study by Page et al. [19], but was lower compared to results from Engel et al., who found that in-hospital mortality of severe sepsis was 51.5% [20].

Our results showed that 75% of patients with septic shock died under admission. A third of the patients with severe sepsis died in the first three months. Forty percent of patients with sepsis of any severity and 46% of patients with severe sepsis died in less than a year after the sepsis event. This supports the hypothesis that the risk of early mortality is still high, especially during the first year after the sepsis event [21]. Long-term survival was shown to be statistically shorter in the group with sepsis of any severity compared with the infection-free group and the infection without sepsis group. Figure 1 shows that the last part of the slopes in the Kaplan–Meier curves seemed to be similar across the infection groups, which could suggest that infectious mortality was a relatively early event, followed by mortality from underlying comorbidities. This is in line with results from the study by Storgaard et al. [8]. Furthermore, survival in patients with infection without sepsis was shorter compared to the infection-free group.

Patients older than 85 years had significantly shorter survival after a sepsis event. Park et al. did not find age to be a significant variable in 28-day mortality [7]. However, advanced age has been shown to be associated with greater mortality rates in other studies [22,23].

Even after adjusting for age and gender, all-cause and short-term risk of death were higher in severe sepsis and septic shock patients than in patients without infection and in patients with infection without sepsis. However, we did not show that the severity of infection was a predictor for long-term mortality in the model adjusted for age and gender. Furthermore, septic shock was not found to be a predictor for long-term mortality. This could be explained by the low number of events in this group.

There are discrepancies between some results from our study and those from other studies. We cannot exclude the significance of definitions and methods that are used for extracting and analyzing data. However, other factors, such as focus, etiology and management of infection/sepsis can influence both outcomes and results [7].

## 5. Conclusions

Our results showed that more severe infection forms tend to influence survival in the first days and months after a sepsis episode. This is more pronounced in the older population. Severity of infection contributed to the increased risk of death in all-cause, in-hospital and 28-day mortality. As for the period from 6 months to 1 year, severity of infection could not, by itself, explain the increased risk of death in patients with infection. Our findings suggest that diagnostic and treatment optimizations are needed regarding management of patients with severer forms of sepsis in order to reduce in-hospital mortality in the Faroe Islands.

## Figures and Tables

**Table 1:** Comparison of demographic and clinical characteristics among patients with infections without sepsis, patients with different sepsis severities and infection-free patients.

Characteristic	Infections without Sepsis		Sepsis		Severe Sepsis		Septic Shock		Infection Free	
**Gender**										
**Male**	142	(47.7)	102	(47.7)	114	(51.1)	12	(60.0)	706	(53.9)
**Female**	156	(52.3)	112	(52.3)	109	(48.9)	8	(40.0)	603	(46.1)
**Age groups**										
**15–64**	108	(36.2)	101	(47.2)	63	(28.3)	6	(30.0)	705	(53.9)
**65–84**	128	(43.0)	92	(43.0)	107	(48.0)	7	(35.0)	510	(39.0)
**85+**	62	(20.8)	21	(9.8)	53	(23.8)	7	(35.0)	94	(7.2)
**Immunosuppression**	84	(28.2)	64	(29.9)	64	(28.7)	3	(15.0)	n.a.	(n.a.)
**Infection focus**										
**Lower respiratory tract**	92	(30.9)	64	(29.9)	81	(36.3)	8	(40.0)		
**Upper respiratory tract**	6	(2.0)	3	(1.4)	4	(1.8)	0	(0.0)		
**Genitourinary tract**	55	(18.5)	44	(20.6)	46	(20.6)	1	(5.0)		
**Abdomen**	8	(2.7)	4	(1.9)	8	(3.6)	2	(10.0)		
**Brain**	1	(0.3)	1	(0.5)	1	(0.4)	0	(0.0)		
**Skin-soft tissue**	23	(7.7)	20	(9.3)	18	(8.1)	1	(5.0)		
**Bone joint**	6	(2.0)	1	(0.5)	2	(0.9)	0	(0.0)		
**Catheter**	5	(1.7)	3	(1.4)	12	(5.4)	3	(15.0)		
**Other infection**	102	(34.2)	74	(34.6)	51	(22.9)	5	(25)		

Data are presented as absolute number (%).

**Table 2:** Cumulative all-cause mortality and in-hospital mortality in patients hospitalized with community-acquired infection of any severity.

Population	Cumulative All-Cause Mortality			In-Hospital Death
	Until 28th Day	Until 180th Day	Total	
**Patients without infections**	(2.1)	(4.2)	(9.4)	(2.0)
	1.4–3.1	3.2–5.5	7.8–11.2	1.3–2.9
**Infections** **without SIRS**	(12.4)	(24.5)	(35.2)	(9.7)
	8.7–17.1	19.2–30.8	28.8–42.6	6.5–14.0
**Sepsis of** **any severity**	(17.5)	(27.8)	(40.5)	(13.6)
	13.9–21.8	23.2–33.1	34.9–46.8	10.4–17.4
**Sepsis**	(9.3)	(19.6)	(31.3)	(7.5)
	5.7–14.4	14.1–26.5	24.3–39.8	4.3–12.1
**Severe sepsis**	(20.2)	(31.4)	(45.7)	(13.9)
	14.7–27.0	24.5–40.0	37.3–55.5	9.4–19.7
**Septic shock**	(75.0)	(75.0)	(80.0)	(75.0)
	50.1–99.9	50.1–99.9	60.2–99.9	50.1–99.9
***p*-value**	<0.001	<0.001	<0.001	<0.001

(%), CIs and *p*-values.

**Table 3:** Characteristics of patients hospitalized with community-acquired infection of any severity who died and those who survived up to 1 year after the admission.

	Died < 28 d		Died 29–180 d		Died ≥ 181 d		Survived	
**Total**	145	(7.0)	110	(5.3)	158	(7.7)	1651	(80.0)
**Gender**								
**Male**	77	(53.1)	52	(47.3)	91	(57.6)	856	(51.8)
**Female**	68	(46.9)	58	(52.7)	67	(42.4)	795	(48.2)
**Age**	77.50 ± 12.45		75.17 ± 13.16		77.13 ± 12.54		59.22 ± 19.59	
**Infection-free**	28	(19.3)	27	(24.5)	68	(43.0)	1186	(71.8)
**Infection**	37	(25.5)	36	(32.7)	32	(20.3)	193	(11.7)
**Sepsis of any kind**	80	(55.2)	47	(42.7)	58	(36.7)	272	(16.5)
**Sepsis**	20	(13.8)	22	(20.0)	25	(15.8)	147	(8.9)
**Severe sepsis**	45	(31.0)	25	(22.7)	32	(20.3)	121	(7.3)
**Septic shock**	15	(10.3)	0	(0.0)	1	(0.6)	4	(0.2)

Data are presented as the absolute number (%) and means.

**Table 4:** Short-term, intermediate-term and long-term mortality in patients hospitalized with community-acquired infections of any severity compared with infection-free patients.

	Unadjusted HR (95%CI)	*p* Value	Adjusted HR ^a^ (95%CI)	*p* Value
**All-cause mortality**				
**Infection free**	Reference group		Reference group	
**Infection without SIRS**	4.452 (3.431–5.778)	<0.001	3.399 (2.610–4.425)	<0.001
**Sepsis**	3.807 (2.827–5.128)	<0.001	3.701 (2.747–4.988)	<0.001
**Severe sepsis**	6.438 (4.949–8.375)	<0.001	4.142 (3.168–5.416)	<0.001
**Septic shock**	27.521 (16.136–46.023)	<0.001	18.660 (10.995–31.670)	<0.001
**Short-term mortality < 28 days**				
**Infection free**	Reference group		Reference group	
**Infection without SIRS**	6.052 (3.704–9.889)	<0.001	4.501 (2.737–7.403)	<0.001
**Sepsis**	4.486 (2.527–7.962)	<0.001	4.159 (2.341–7.389)	<0.001
**Severe sepsis**	10.366(6.466–16.616)	<0.001	6.779 (4.185–10.979)	<0.001
**Septic shock**	71.724 (38.110–134.988)	<0.001	46.985 (26.687–89.420)	<0.001
**Intermediate-term mortality < 180 days**				
**Infection free**	Reference group		Reference group	
**Infection without SIRS**	7.014 (4.258–11.553)	<0.001	5.529 (3.338–9.158)	<0.001
**Sepsis**	5.707 (3.257–10.020)	<0.001	5.512 (3.137–9.685)	<0.001
**Severe sepsis**	7.013 (4.070–12.083)	<0.001	4.921(2.833–8.550)	<0.001
Septic shock *	-	-	-	-
**Long-term mortality ≥ 181 days**				
**Infection free**	Reference group		Reference group	
**Infection without SIRS**	2.720 (1.787–4.141)	<0.001	2.110 (1.381–3.225)	0.001
**Sepsis**	2.801 (1.771–4.430)	<0.001	2.885 (1.823–4.566)	<0.001
**Severe sepsis**	4.387 (2.882–6.680)	<0.001	2.631 (1.715–4.037)	<0.001
**Septic shock**	4.525 (0.628–32.621)	0.134	2.914 (0.403–21.077)	0.289

HR—hazard ratio, 95%CI—95% confidence interval, ^a^ Multivariable Cox regression analysis including sex and age, * HRs were not calculated in the septic shock category because of the small number of patients.

## Data Availability

The datasets used and/or analyzed during the current study are available from the corresponding author on reasonable request.
